# The New Totalitarians: The Swedish COVID-19 strategy and the implications of consensus culture and media policy for public health

**DOI:** 10.1016/j.ssmph.2021.100788

**Published:** 2021-04-06

**Authors:** Martin Lindström

**Affiliations:** Social Medicine and Health Policy, Department of Clinical Sciences in Malmö, Lund University, S-205 02, Malmö, Sweden

**Keywords:** Pandemic, Coronavirus, COVID-19, Swedish strategy, Herd immunity, Individual responsibility, Evidence based medicine, Frode Forland, Mass media, Consensus culture, Postmodernism, Sweden

## Abstract

**Aims:**

The aim is to discuss implications of consensus culture in combination with media policy in Sweden with regard to the Swedish COVID-19 strategy in the spring of 2020.

**Methods:**

Investigation of prerequisites for scrutiny by the Swedish mass media of the Swedish strategy in the spring of 2020 based on discussion regarding consensus culture, media structure and postmodernism in science, politics and administration.

**Results:**

The Swedish strategy entailed strong initial confidence in herd immunity (although not officially stated), individual responsibility, evidence based medicine and substantial neglect to cooperate internationally. The strategy may be regarded partly as a result of the postmodern view of science and society predominant in Sweden. A tradition of top down consensus culture combined with mass media's financial and partly structural dependence of the state may help explain the comparative lack of critical questions regarding the strategy at the press conferences in the spring of 2020.

**Conclusions:**

Mass media in Sweden should become more financially and structurally independent of the state. The reporting by Swedish media in the spring of 2020 should be subject to peer-reviewed research.

## Introduction

Sweden has long been praised for internationally high levels of income equality and internationally good population health and high life expectancy (Wilkinson & Pickett, 2010). However, the COVID-19 pandemic and the Swedish response to the pandemic has at least partly changed the picture of Sweden as a beacon of equality and good public health. The official aim of the initial Swedish strategy was to reduce the spread of the coronavirus in order not to strain the Swedish health care system beyond its capacity, while at the same time protecting the chronically ill and the elderly. The strategy also included the aims to reduce the impact on functions of importance in society, to reduce consequences for members of the public and companies, to reduce anxiety and to implement the right measures at the right time (Regeringskansliet, 2020). The strategy involved fewer and often looser restrictions than in the closest neighboring countries Denmark, Norway and Finland (see description in Lindström, 2020a). The government and the Public Health Agency (*Folkhälsomyndigheten*) denied the proposition that the Swedish strategy involved the goal to achieve natural herd immunity. However, the Swedish state (public service) television reporting was overwhelmed with reporting regarding the rapidly approaching achievement of natural herd immunity in the spring of 2020. First, it was reported that half the population of Sweden would be expected to be infected at the end of April 2020 (SVT, 2020a). This would have resulted in the achievement of natural herd immunity in a very short time, because the official narrative also included the notion that natural herd immunity would be achieved when between 50% and 60% of the population had been infected (SVT, 2020b). Later, when it became obvious that these calculations for April had been completely incorrect, the official narrative lowered the level necessary to achieve herd immunity to 40% (SVT, 2020c), which may be sufficient to achieve natural herd immunity under certain preconditions including population heterogeneity (Britton et al., 2020). Even later, public access to the e-mails within the Public Health Agency revealed that the official answer that the strategy did not consider the possibility of natural herd immunity had not been accurate (Anderberg, 2020). Some other specific traits of the Swedish strategy included a strong belief in individual responsibility in handling the pandemic, thus indirectly diminishing the responsibility of the government, national politicians and the Public Health Agency. It also included a strong emphasis on evidence based medicine with regard to any protective actions or measures such as mouth protection in the spring of 2020. In sharp contrast, it included no corresponding strong demands to utilize the experience from earlier hit countries that had initially succeeded in limiting the spread (e.g. South Korea) or countries that had failed (e.g. Italy). It seems that the Public Health Agency paradoxically defined high standards from evidence based medicine regarding protective measures but not regarding the strategy itself (Lindström, 2020a). Finally, there was also a relative neglect compared to other countries to follow recommendations from the WHO and to cooperate with the neighboring Nordic countries and the EU (Standard Ethics, 2020). In the spring of 2020, a number of initially 22 researchers repeatedly criticized, the seeming goal to achieve natural herd immunity and the high death tolls (Carlsson et al., 2020; Bjermer et al., 2020). The Public Health Agency just denied that natural herd immunity was involved in the Swedish strategy.

The results of the Swedish strategy have so far substantially differed from the other Nordic countries. The per capita death toll did not differ between Denmark and Sweden during the first three weeks after the implementation of the Danish restrictions in mid-March 2020. This may indicate similar spread in Denmark and Sweden until mid-March (Lindström, 2020a). Still, by the end of June 2020 the number of deaths with COVID-19 was 5310 in Sweden (10.3 million inhabitants), 605 in Denmark (5.8 million inhabitants), 249 in Norway and 328 in Finland (World Health Organization, 2020). This first wave did not only have adverse effects on public health in general, but it also had adverse effects on health equality and equity. A report from Region Stockholm, the largest and most hard hit region with responsibility for the healthcare system, showed that the population segments with lower income, lower education and lack of possibility to work at home had significantly higher risk of hospital care and death until 30 June. The analyses also revealed higher mortality among particularly the foreign-born groups from Somalia, Libanon, Syria, Turkey and Estonia (Bartelink et al., 2020). In the aftermath of the present second wave in Sweden, the total death toll in COVID-19 reported to the WHO from the start of the pandemic was 13,146 deaths in Sweden, 2390 in Denmark, 786 in Finland and 639 in Norway until 16 March 2021 (with some delay in the WHO statistics) (World Health Organization, 2021). By then, the total death rates per 100,000 inhabitants were 130.2 in Sweden, 41.3 in Denmark, 14.2 in Finland and 11.8 in Norway. The corona commission concluded at the end of 2020 that the high death toll among the elderly in care centers could be explained by the high general spread of the contagion in society (Melin et al., 2020). The government, the Public Health Agency, the regions and the municipalities responsible for the care of the elderly subsequently spent later parts of 2020 and early 2021 blaming each other for the development. This blame game partly took transparency, inclusiveness and accountability included in WHO:s ethical pandemic principles out of play.

In an interview in the daily newspaper *Svenska Dagbladet* in mid-May 2020 Frode Forland, the Norwegian equivalent to the Swedish state epidemiologist, not only criticized the initial Swedish strategy to handle the pandemic. He also posed the question why the Swedish mass media did not ask substantially critical questions regarding the strategy and high death tolls during the daily press conferences with the Public Health Agency in the spring. Forland stated that this lack of relevant critical questions was a surprise to him, and that it should be regarded as a serious problem (Falkirk, 2020). Although a few voices from the press criticized the strategy already in mid-March (Wolodarski, 2020), critical Swedish press voices were mostly absent from the official daily press conferences during the spring of 2020 (Andersson et al., 2021). The critical questions predominantly came from international journalists. Sveriges Radio even asked the question “who is the German putting pressure on the Public Health Agency?” at the press conferences (Studio Ett, 2020). Clearly, the German journalist from the German TV channel ARD was regarded as a remarkable specimen. Substantially later, this had changed to an important extent following the recurring unexpectedly high death tolls in the second wave beginning in late autumn 2020. Still, the Swedish state television (SVT) initially sought to omit the King's criticism of the strategy from their interview a week before Christmas in 2020 (BBC News, 2020; Nordberg, 2020), but the King insisted it should be expressed in the interview and reported.

The aim is to investigate and discuss top down consensus culture combined with media policy as prerequisites for mass media reporting of the Swedish strategy in the spring of 2020.

## Methods

The fact that Sweden is the most postmodern society in the world and the recent dominance of postmodern ideology, view of science and culture among decision makers in Sweden has been suggested as one plausible underlying precondition for the Swedish strategy (Lindström, 2020a). In short, the postmodern view of science tends to reject the notions that objective truths exist in science and that experiences accumulated across time are generally valid until disproved. Postmodernism mostly opposes scientific and other sources of authority. Instead, new “truths” can be created in science based on standpoints that sometimes partly or even completely neglect empirical observations and practical experiences (Inglehart, 1997). The prerequisites for the main task of the mass media to scrutinize political and administrative decision makers will be highlighted in light of Swedish consensus culture and Swedish media policy. Peer-reviewed references and mass media will be scrutinized to provisionally highlight these prerequisites.

## Results

In 1971, the British journalist Roland Huntford, who had been stationed as a foreign country affairs correspondent and journalist in Sweden in the late 1960s and early 1970s, published his book “The New Totalitarians” (1971) characterizing the political, mass media and corporate culture in Sweden. Partly inspired by Aldous Huxley's “Brave New World” (Huxley, 1932/2004), Huntford describes a country permeated by political, mass media, cultural and corporate top down consensus culture, a consensus verging and balancing on the close border of totalitarianism. One example given by Huntford concerns an agreement between a local company and its employees, which resulted in higher salaries for the specific employees employed by the local company than the collective agreement achieved in the national negotiations between the corresponding central employers' association and labor union. The dissatisfied central labor union could not turn to its own local section in order to cancel the local agreement. This would naturally have appeared disloyal. Instead, the central labor union turned to its adversary the central employers' association in order to make it encourage the local employer to legally dissolve the local agreement. Huntford also interviewed the director of the second channel of the Swedish state television (then a state TV monopoly with two TV channels) who stated that “TV would never attack the Prime Minister and government, because the average Swede identifies himself with the State and with the corporations that exercise political influence. So TV feels part of the State.” Another state television official admitted to Huntford that “the aim of the television service is to persuade the Swedes that they live in the best of all worlds” (Huntford, 1971).

Huntford concluded that this top down consensus mentality permeates most or all sectors of Swedish society. It should be noted that the kind of coercion Huntford refers to does not result in imprisonment, deportations, exiling or executions based on political opposition or other forms of dissent. Instead, the mechanisms of coercion include e.g. voluntary social control, social ostracism, social isolation, vilification and firing people from their jobs, if possible. Huntford denoted such processes as the practice of “soft totalitarianism”. There are no signs that this political culture has been weakened in Sweden in the subsequent half-century. In 2013, Henric Ekengren Oscarsson, political science professor at Göteborgs Universitet, coined the notion of “the opinion corridor” (*åsiktskorridoren*), to describe a Swedish public debate climate characterized by the very narrow limits of viewpoints possible to express on many political and cultural issues (Skata et al., 2016). Swedish mass media had to some extent come to serve as a gatekeeper for the establishment, rather than a scrutinizer of political and administrative decision makers. The observations by Huntford are in some ways similar to those forwarded by Christopher Lasch in “The Revolt of the Elites and the Betrayal of Democracy” (1995) that postmodern political and other elites have betrayed the bottom up aspect of democracy in western countries since the 1960s/1970s and onwards (Lasch, 1995).

Following particularly the second wave in Sweden in late autumn 2020, some Swedish editors and journalists criticized the comparative lack of critical questions from Swedish journalists at the press conferences with the Public Health Agency in the spring of 2020 in retrospect. Andén (2020) suggested in June that Swedish journalists had something substantial to learn from “the crazy German”. Teodorescu Måwe (2020) concluded that the role of Swedish mass media in the spring of 2020 should be thoroughly scrutinized, and asked the rhetorical question if it was just by chance that the most critical questions came from a German journalist? Croneman (2020) suggested that critical voices with a serious message should be let into public debate and that their viewpoints should be publicly scrutinized. In March 2021, a report released from the Institute for Media Studies showed that it was foreign journalists which had asked the most critical questions in the spring. Swedish journalists had to an important extent focused on “informing the population”, which delayed critical scrutiny (Andersson et al., 2021). A report from *Myndigheten för Samhällsskydd och Beredskap* and Göteborg University only scrutinized three Swedish newspapers. However, “the German journalist” is mentioned, and the authors ask how long it is acceptable for the media to strive for national unity instead of asking critical questions (Ghersetti & Odén, 2021)?

The top down consensus culture is to an important extent complemented with mostly indirect financial and structural control over mass media. Until the end of 1987, there was a complete state monopoly for television in Sweden, and only one TV channel existed. In 1969, a second TV channel started. On January 1, 2019, the system with TV licenses paid by owners of television sets in order to finance Swedish state (public service) television was replaced by a mandatory tax to finance state television (SVT) and radio broadcasting (Sveriges Radio). This tax is paid by all adult tax payers. This idea that political decision makers make budget decisions regarding the mass media supposed to scrutinize the same office holders was heavily criticized (Rudbeck, 2018) and still remains problematic. Furthermore, the state also increasingly finances most daily morning newspapers in Sweden with tax money, originally for reasons of falling numbers of subscribers and falling circulation. Many morning newspapers would presently not survive without government state grants. An additional sum of further 200 million SEK was granted to the morning newspapers at the beginning of the implementation of the Swedish strategy in March 2020 (Tidningarnas Telegrambyrå, 2020).

The issue of indirect control over mass media and communication is greater and more internationally relevant than just financial influence over important national Swedish TV channels and newspapers. Starting in 2017, the Social Democratic government in Sweden has granted tax exceptions to the global US-based social media companies. Profits approximating 5.5 billion SEK are taxed at rates of single percent units. These global social media companies have also been granted a heavily reduced energy tax compared to other consumers. As a consequence, individual consumers and many other companies now pay a 90 times higher energy tax than the richest international companies in the world (Alestig, 2014; Aftonbladet, 2020a; Granlund & Svensson, 2020). The strategical considerations regarding information control behind these tax exceptions will be discussed in the discussion section.

## Discussion

This presentation does not suggest that critical questions are prohibited in Sweden. Sweden has its own constitutional law that formally regulates free speech- *Yttrandefrihetsgrundlagen (YGL)* (1992). Together with another constitutional law, *Tryckfrihetsförordningen* (1949), which concerns the freedom to print and publish, it also regulates mass media in Sweden. This short communication suggests that a combination of postmodern view of science, top down consensus culture and mass media with strong direct (SVT-the state television and Sveriges Radio) and indirect (e.g. daily newspapers) dependence on government for financial survival all plausibly contributed to a comparative lack of scrutiny of the strategy by the mass media in the spring of 2020. It also suggests that a mass media environment lacking critical voices scrutinizing the political and administrative decision makers may in certain situations such as the handling of the COVID-19 pandemic have negative effects on public health. Regarding most topical issues, the effects of the consensus culture may more or less easily be hidden behind a veil of rhetoric and partial one-sidedness in public discussion. However, in light of high death tolls, high infection rates and yet unknown consequences of long-term illness following the pandemic, the consequences of the consensus culture make the one-sidedness of mass media in the spring of 2020 more apparent than usual. This short communication also suggests that anyone with a monopoly of information, including a government and a public authority such as the Public Health Agency, can be responsible for miscalculations, misinterpretations, misunderstandings and even self-contradiction (e.g. regarding herd immunity). Such public authorities would most probably benefit from more intensive scrutiny already at an early stage. Forland has recently testified that in Sweden in the early part of the pandemic decisions were made by fewer people and following less discussion than in Norway and Denmark (Jakobsen & Amundsen, 2021). Paradoxically, coercion may be the antithesis to traditional scientific authority, because postmodern “constructed” truths may have to be protected from scrutiny and discussion.

Already in April 2020, a Swedish peer-reviewed article investigating “misinformation” in international mass media regarding the Swedish strategy such as the narratives Sweden has a herd immunity strategy, Sweden is not following expert advice and Sweden is not following WHO recommendations was submitted and somewhat later published. It concludes that “While these narratives are partially grounded in reality, the language and examples used to frame the story distorted the accuracy of the reporting”. The article itself starts with the following opening statement in the abstract: “In the first month of the 2020 COVID-19 pandemic, Sweden took the same strategy as most other countries, working to “flatten the curve”, by slowing transmission so that the healthcare system could cope with the disease” (Irwin, 2020a). This opening statement is not correct. Many countries in Europe and other parts of the world aimed to eradicate the contagion by social distancing, social isolation, and most importantly continued mass testing and contact tracing. Sweden stopped mass testing and contact tracing in the second week of March 2020 following an active recommendation to all regions by the Public Health Agency to only test in-hospital patients and specific risk groups. This policy was swiftly implemented even in regions with low infection rates in the spring (Karlsson, 2020). The opening statement is also contradicted by the testimony of the masterminds behind the Swedish strategy themselves. In early 2020 the former state epidemiologist (1995–2005), professor Johan Giesecke, was contracted as senior consultant by the Public Health Agency. Giesecke was certainly at least one of the masterminds behind the strategy. On April 3, 2020, Giesecke stated that “all other countries are doing wrong”, and predicted that Sweden would return to close to “normalcy” at some point in May 2020 following transmission across the population, because that was his “stomach feeling” (Grundberg Wolodarski, 2020). These observations lead to three interesting questions. First, why make an opening factual statement that is factually wrong in an article investigating “misinformation” in the international mass media? Second, why scrutinize the international mass media reporting of the Swedish strategy in this very early part of the pandemic? If the initial Swedish strategy had been closely similar to most other countries, there would probably have been no need for such scrutiny anyway. Third, why did neither the author of the article nor any other Swedish public health academics scrutinize Swedish mass media reporting of the Swedish strategy in the early spring 2020? Such a scrutiny would certainly have been motivated given headlines such as “the deceptive mortality of the coronavirus”, comparing the COVID-19 pandemic with a seasonal influenza (Sydsvenskan, 2020), and the repeated reporting of total number of infected required to achieve natural herd immunity (SVT, 2020; SVT, 2020b; SVT, 2020c). Scrutiny would also have been motivated following the contradiction between the official denial of herd immunity as a strategic goal versus the testimony by Giesecke (Grundberg Wolodarski, 2020).

Politics and mass media have in recent years showed increased interest in “the Sweden picture” (*Sverigebilden*) (Irwin, 2019; Aftonbladet, 2020b). Attempts were also made to correct the image of the Swedish strategy conveyed by e.g. international scientists (Irwin, 2020b). The need for an official “Sweden picture” has appeared because debate is restricted to an important but varying extent in several political, cultural and social issues, i.e. “the opinion corridor”. Self-imposed restrictions on debate often lead to suboptimal decisions that adversely affect real life experiences. Recent developments have also shown the public service companies to be prone to scrutinize individuals and groups of researchers seemingly to a higher extent than to scrutinize the public office holders in the spring of 2020 (Sveriges Radio, 2021).

The consensus culture described by Huntford probably has historical roots in a socially cohesive society. This society has previously been described as well functioning, and the effectiveness of public institutions and companies praised. A combination of the protestant work and duty ethic (Weber, 1905/2001) and the belief in science and practical experiences resulted in a highly efficient society. Why has this consensus culture backfired? Why has the need for a “Sweden picture” emerged? A plausible part of the answer spells postmodernism. The postmodern view of science, truth and inclination to ignore real life experiences in favor of wishful ideological and utopian thinking may have a particularly devastating effect if it comes to dominate in a consensus culture.

Swedish mass media reporting concerning the Swedish strategy in the spring of 2020 should be scientifically investigated. Ideological and financial pluralism in the mass media may promote discussion which may also be benevolent for public health. Media policy should promote an ideological multitude of voices supported by a multitude of financial sources. The mechanisms behind tendencies towards “soft totalitarianism” should be scrutinized. The postmodern culture and view of science and its official status in public administration and public life in Sweden (Lundberg, 2020) should be questioned and discussed.

The global US-based social media companies have been granted tax exceptions and economic advantages in Sweden since 2017 to an extent that approximately corresponds to those granted to the nobility in France before the French revolution in 1789. Why would a Social Democratic government grant such economic privileges? Giving such tax exceptions economically promoting the super richest in the world would seem as violently opposing the essence of Social Democratic ideology (Lindström, 2020b). The aim of these tax exceptions has been to attract the building of extremely energy consuming server halls in Sweden. The official argument has been that server halls create jobs. However, very few jobs have been created because such server halls are not labor intensive. Instead, one plausible explanation may be a strategic strive for economic and ideological symbiosis between government and international social media companies in controlling information.

A solution may be new laws which put a fine on censorship of statements in social media that do not violate Swedish law. Such laws are already being processed in several countries, but such measures need further debate.

## Conclusions

A top down consensus culture, a postmodern view of science combined with mass media's financial dependence of the state may help partly explain the comparative lack of critical questions regarding the Swedish strategy in the mass media and at press conferences with the Public Health Agency in the spring of 2020. Mass media in Sweden should become more financially independent of the state. Social media should be stripped from its economic privileges.

## Funding

This short communication has no external funding sources.

## Author statement

This manuscript has not been submitted to any other journal, and is thus not under review in any other journal. The manuscript is the author's original work.

## Ethical considerations

No study participants were included.

## Declaration of competing interest

None.
